# DAXX modulates human papillomavirus early gene expression and genome replication in U2OS cells

**DOI:** 10.1186/s12985-015-0335-z

**Published:** 2015-07-07

**Authors:** Piia Kivipõld, Liisi Võsa, Mart Ustav, Reet Kurg

**Affiliations:** Institute of Technology, University of Tartu, Nooruse 1, 50411 Tartu, Estonia

**Keywords:** Papillomavirus, Transcription, Replication, Virus-host interaction, DAXX

## Abstract

**Background:**

The human papillomavirus (HPV) genomes can replicate, and are maintained as autonomously replicating extrachromosomal plasmids in human U2OS cells. Previous studies have shown that HPV genomes are transcriptionally active in U2OS cells and can express the viral early proteins required for initiation and establishment of HPV replication. In the present work, we have examined the involvement of cellular DAXX protein in HPV replication in U2OS cells.

**Methods:**

We have used indirect immunofluorescence and FISH analysis in order to study HPV replication compartments in U2OS cells. In addition, we have used siRNA knock-down for examining the effect of the DAXX protein on HPV replication and transcription in U2OS cells.

**Results:**

We show that a portion of HPV replication foci are partially co-localized with components of ND10, cellular DAXX and PML proteins. In addition, we demonstrate that the knock-down of the cellular DAXX protein modulates the HPV genome replication and transcription in U2OS cells – papillomavirus replication is reduced in the absence of this component of ND10.

**Conclusions:**

The DAXX protein modulates the early gene expression and the transient replication of HPV genomes in U2OS cells.

## Background

Papillomaviruses are small, double-stranded DNA viruses which infect the epithelial tissue of a wide variety of vertebrates and induce proliferative lesions of the skin and mucosa. The human papillomavirus (HPV) high-risk types, such as HPV16, 18 and 31, are associated with anogenital cancer, and are the primary aetiological agents for cervical carcinoma. Low-risk types, for instance HPV6 or 11, can cause genital warts [1]. Papillomaviruses establish long-term persistent infection in stratified squamous epithelium. The viral life cycle is tightly linked to the differentiation status of the host cells. After infection of the basal cells of the epithelium, the viral genome is replicated until an optimal copy number is reached. This is called the initial amplification, or establishment phase, of PV replication. It is followed by a maintenance phase, during which a constant copy number of extrachromosomal viral genomes is maintained in the nuclei of host cells as they divide. The viral E1 and E2 proteins, together with the host cell replication machinery, are responsible for HPV DNA replication at both early stages of virus replication [reviewed in [2].

Many DNA viruses replicate their genomes in close proximity to ND10. It has been suggested that ND10 is important for antiviral defense and is a part of the intrinsic immune system [3]. Swindle and others have demonstrated that HPV11 replication compartments partially overlap with PML in C33A cells [4]. However, the functional role of ND10 in papillomavirus infection is still controversial. In the case of BPV1, ND10 seems to positively affect papillomavirus infectivity as the presence of PML and intact ND10 are associated with enhanced BPV1 early gene expression [5]. Yet others have shown that PML and ND10 are not required for HPV DNA replication in transfected cells [6]. More recent data show that ND10 proteins have opposing roles in viral transcription; SP100 knock-down enhances HPV18 early transcription in primary human keratinocytes, while PML and DAXX knock-down decreases it [7].

One component of the ND10 is the DAXX protein, which is a highly conserved nuclear protein involved in regulation of apoptosis and gene expression [8]. It is predominantly located in the cell nucleus, where it is associated with two nuclear domains: the ND10 and condensed heterochromatin [9]. In transcription regulation, the DAXX protein functions mainly as a co-repressor. It associates with histone deacetylases (HDACs), DNA methyltransferases (DNMTs), core histones, DEK, ATRX and other chromatin-associated factors [10–13], suggesting that it represses transcription through modification of chromatin. The repressive function of DAXX has been associated with cellular intrinsic immune response against incoming viruses. Through the action of HDACs, DAXX silences immediate-early (IE) gene expression of human cytomegalovirus (HCMV) by inducing a transcriptionally inactive chromatin state around the major IE promoter [3, 14, 15]. The DAXX protein is also involved in negative regulation of adenovirus (Ad5) replication [16], and initiation and maintenance of retroviral silencing, which is facilitated by HDACs and DNMTs recruitment to retroviral (ASV-1, HIV-1) DNA [13, 17, 18].

Most established human cell lines fail to support HPV genome replication. However, both alphapapillomaviruses, including HPV11 and 18, and betapapillomaviruses are able to replicate extrachromosomally and establish a stable replication in the human osteosarcoma cell line U2OS [19]. U2OS cells are not the natural host cells of papillomaviruses, but these cells appear to contain all the necessary cellular factors required for the replication of papillomavirus genomes, and therefore provide a cost-effective system for studying the fundamental processes of papillomavirus replication. In our present work, we have examined the HPV replication foci in U2OS cells in relation to ND10, and the effect of one component of ND10, the DAXX protein, on HPV genome replication and transcription. We show that the DAXX protein modulates the early gene expression and the transient replication of HPV genomes in these cells.

## Results

### HPV replication foci in U2OS cells

The replication of HPV genomes occurs within the distinct replication compartments in the nuclei of U2OS cells [19]. Both E1 and E2 proteins are required for the initiation of HPV replication within the cells [20] and are shown to localize in the HPV replication centers [21]. In this study, we have used the mouse monoclonal antibodies against HPV11 E2 and rabbit polyclonal antibodies against HPV18 E1 in order to characterize the HPV replication foci in U2OS cells by indirect immunofluorescence analysis. These antibodies allow us to detect the corresponding HPV proteins expressed by the HPV minicircle genomes.

**Fig. 1:**
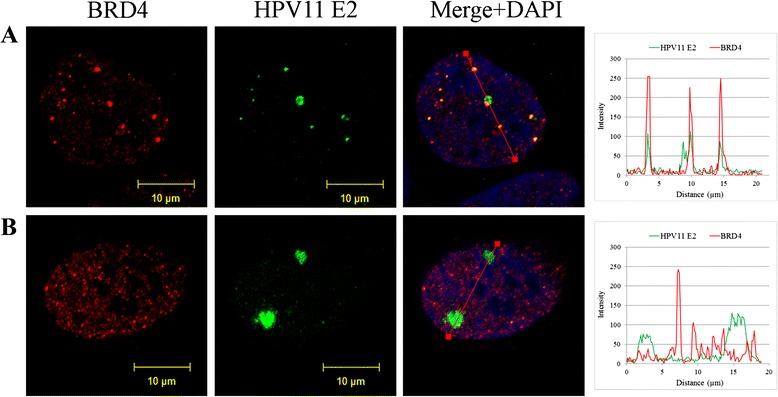
HPV replication foci in U2OS cells. Immunofluorescence analysis of U2OS cells transfected with HPV11 genome. U2OS cells were transfected with wt HPV11 minicircle genomes and grown on coverslips. Six days after transfection cells were fixed and immunostained with antibodies for HPV11 E2 (green) and BRD4 (red). Cell nuclei were detected by DAPI. Analysis was carried out at least three times starting from cell transfections. **a** and **b** represent cells with different number and size of HPV11 E2 foci. On the right panels, the intensity profiles are shown

First, we analyzed the involvement of cellular BRD4 protein in HPV replication in U2OS cells. BRD4 localizes to, and is essential for the formation of HPV replication foci in primary human foreskin keratinocytes. However, when E1 and E2 begin to amplify the viral DNA, BRD4 is displaced from the foci [21]. In order to test whether this is also the case in U2OS cells, we performed an immunofluorescence assay. Six days after transfection with 2 μg of wt HPV11 minicircle genomes, the cells were fixed and immunostained for HPV11 E2 and BRD4 proteins. As shown in Fig. 1, the individual U2OS cells contained different number of HPV11 E2 foci (Fig. 1 HPV11 E2 column). We detected up to ten HPV11 E2 foci, with an average value of four foci per cell nucleus. The HPV replication foci also had a different size. The small HPV11 E2 foci co-localized with the cellular BRD4 protein (Fig. 1a), and were reminiscent of the small early HPV replication foci that were described in human keratinocytes by Sakakibara and others [21]. Large HPV11 E2 foci were also visible in U2OS cells at the same time-point, and the cellular BRD4 protein was absent from these foci (Fig. 1b). Overall, this indicates the involvement of BRD4 in the early steps of replication foci formation in U2OS cells, similar to what has been observed in human keratinocytes [21].

### HPV replication foci localization in relation to ND10 in U2OS cells

**Fig. 2:**
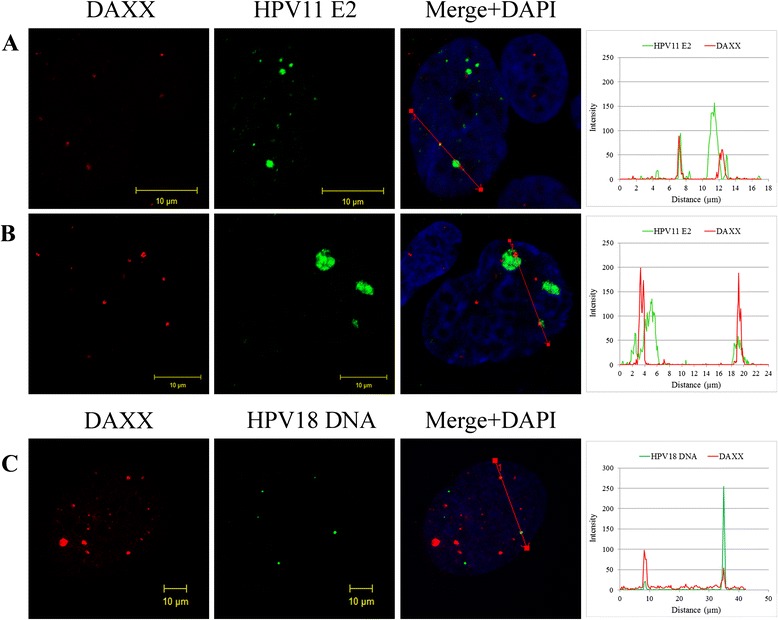
The localization of HPV replication foci in U2OS cells in relation to the DAXX protein. **a** and **b** Immunofluorescence analysis of U2OS cells transfected with HPV11 genome. U2OS cells were transfected with wt HPV11 minicircle genomes and grown on coverslips. Six days after transfection cells were fixed and immunostained with antibodies for HPV11 E2 (green) and DAXX (red) proteins. Cell nuclei were detected by DAPI. Analysis was carried out at least three times starting from cell transfections. **a** and **b** represent cells with different number and size of HPV11 E2 foci. **c** FISH analysis of U2OS cells transfected with HPV18 genome. U2OS cells were transfected with wt HPV18 minicircle genomes and grown on microscope slides. Six days after transfection cells were fixed and combined immunofluorescence and FISH analysis was performed. DAXX signal is visible in red and HPV18 DNA in green. Cell nuclei were detected by DAPI. Analysis was carried out at least three times starting from cell transfections. On the right panels, the intensity profiles are shown

The main aim of this work was to analyze the localization of HPV replication centers in relation to components of ND10 in U2OS cells. Many DNA viruses replicate their genomes in close proximity to ND10, and several reports link multiple stages of HPV infection with these nuclear sites [4, 5, 22, 23]. However, the role of ND10 in papillomavirus infection is still controversial. In order to analyze the link between HPV replication and ND10 in U2OS cells, we first analyzed the localization of the HPV E2 protein and cellular DAXX protein, a constitutive component of ND10. For this purpose, U2OS cells were transfected with 2 μg of wt HPV11 minicircle genomes, fixed six days later and immunostained for HPV11 E2 and cellular DAXX proteins. On average, seven DAXX-containing foci per cell nucleus were observed (Fig. 2 DAXX column), and 34 % of the E2 foci overlapped partially with DAXX-containing foci. Representative images of cells with different number and size of HPV11 E2 foci and DAXX staining are shown in Fig. 2a and b. A similar analysis with HPV18 replication centers failed due to the fact that we were unable to find mouse monoclonal antibodies against the DAXX protein suitable for immunofluorescence analysis. Therefore, we performed immunofluorescence analysis coupled with FISH detection of HPV18 DNA. U2OS cells were transfected with 5 μg of wt HPV18 minicircle genomes and fixed six days later. The cells were immunostained for DAXX protein and HPV DNA was detected with an HPV18 specific probe (Fig. 2c). We observed at least partial co-localization of DAXX and HPV DNA in 22 % of the HPV18 DNA foci in U2OS cells.

**Fig. 3:**
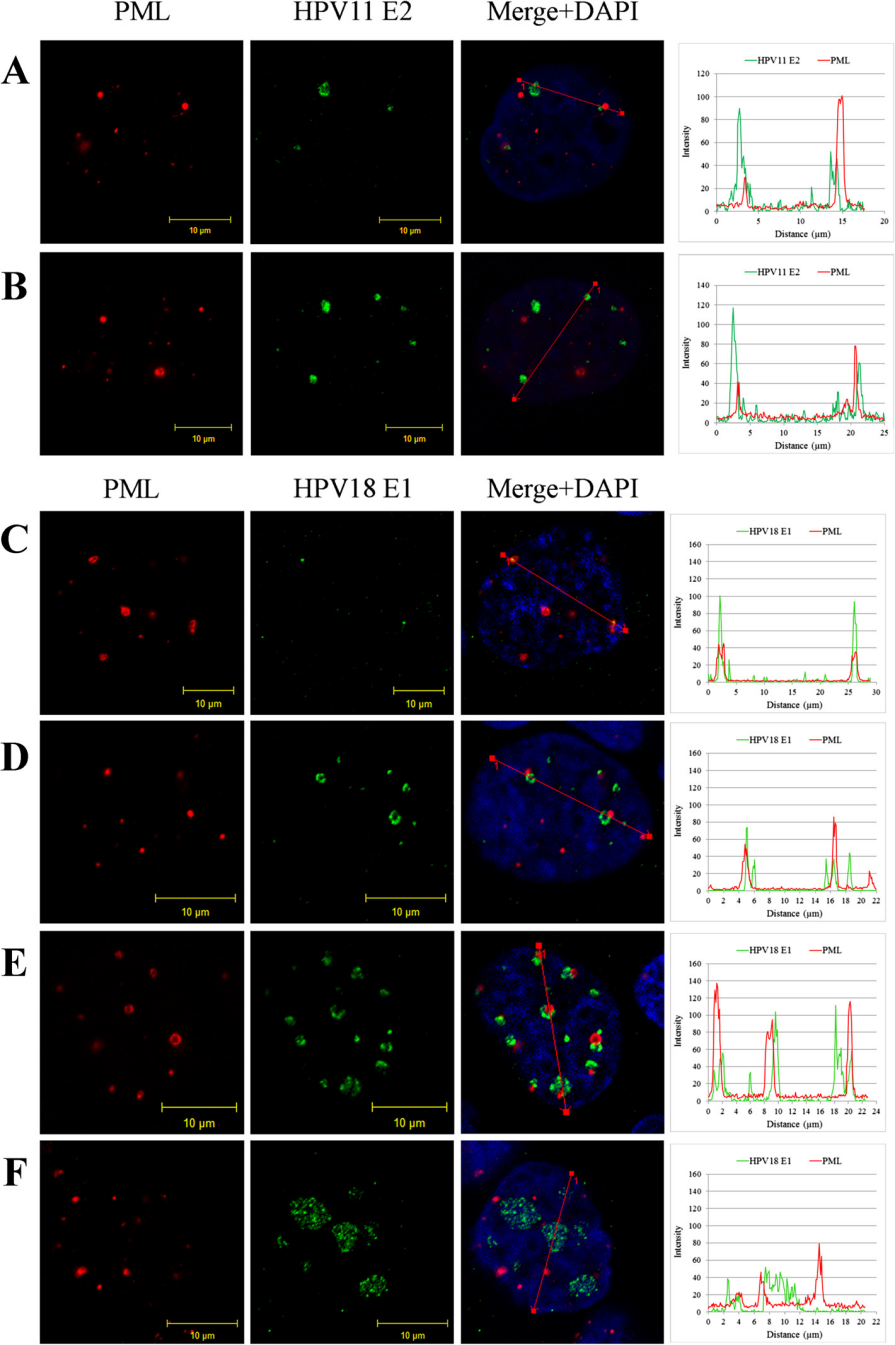
The localization of HPV replication foci in U2OS cells in relation to the PML protein. Immunofluorescene analysis of U2OS cells transfected with HPV11 and HPV18E8- genomes. **a** and **b** U2OS cells were transfected with wt HPV11 minicircle genomes and grown on coverslips. Six days after transfection cells were fixed and immunostained with antibodies for HPV11 E2 (green) and PML (red) proteins. Cell nuclei were detected by DAPI. Analysis was carried out at least three times starting from cell transfections. **a** and **b** represent cells with different number and size of HPV11 E2 foci. **c**, **d**, **e** and **f** U2OS cells were transfected with HPV18E8- minicircle genomes and grown on coverslips. Six days after transfection cells were fixed and immunostained with antibodies for HPV18 E1 (green) and PML (red) proteins. Cell nuclei were detected by DAPI. Analysis was carried out at least three times starting from cell transfections. **c**, **d**, **e** and **f** represent cells with different number and size of HPV18 E1 foci. On the right panels, the intensity profiles are shown

In order to confirm the partial co-localization of HPV replication centers and ND10, immunofluorescence analysis was performed with another component of ND10, the PML protein. First U2OS cells were transfected with 2 μg of wt HPV11 minicircle genomes and immunostained for HPV11 E2 and cellular PML proteins. The PML signal was observed in a punctate pattern in the cell nucleus, with an average of 14 PML structures per nucleus (Fig. 3a, b; PML column). 44 % of the E2 foci overlapped partially with PML-containing foci. In many cases, HPV replication foci and ND10 localized side by side. Representative images of cells with different number and size of HPV11 E2 foci and PML staining are shown in Fig. 3a and b. A similar analysis was performed with HPV18; however, in this case the immunofluorescence analysis was carried out with HPV18E8- genome. The HPV18E8- mutant replicates at a higher level than the wt HPV18 genome [24, 25], resulting in a higher and more easily detectable HPV18 E1 protein level in the transfected cells. In the case of the HPV18E8- genome, we detected up to 24 E1 foci per cell nucleus, with an average of 10 foci per cell. Examples of typical cells with different sizes of E1 foci are shown in Fig. 3c, d, e and f. 42 % of the E1 foci overlapped, at least partially, with PML signal in U2OS cells six days after transfection. However, in most of the cells, there was no perfect co-localization of HPV18 E1 protein and PML signals; rather, a portion of the E1 foci overlapped with PML partially. In a few cells, we were able to detect very small E1 foci surrounded by the PML protein, as shown in Fig. 3c.

In summary, our immunofluorescence analyses indicate that a fraction of HPV replication foci localize in proximity of components of ND10, the DAXX and PML proteins in U2OS cells.

### DAXX modulates the transient replication of HPV genomes in U2OS cells

**Fig. 4:**
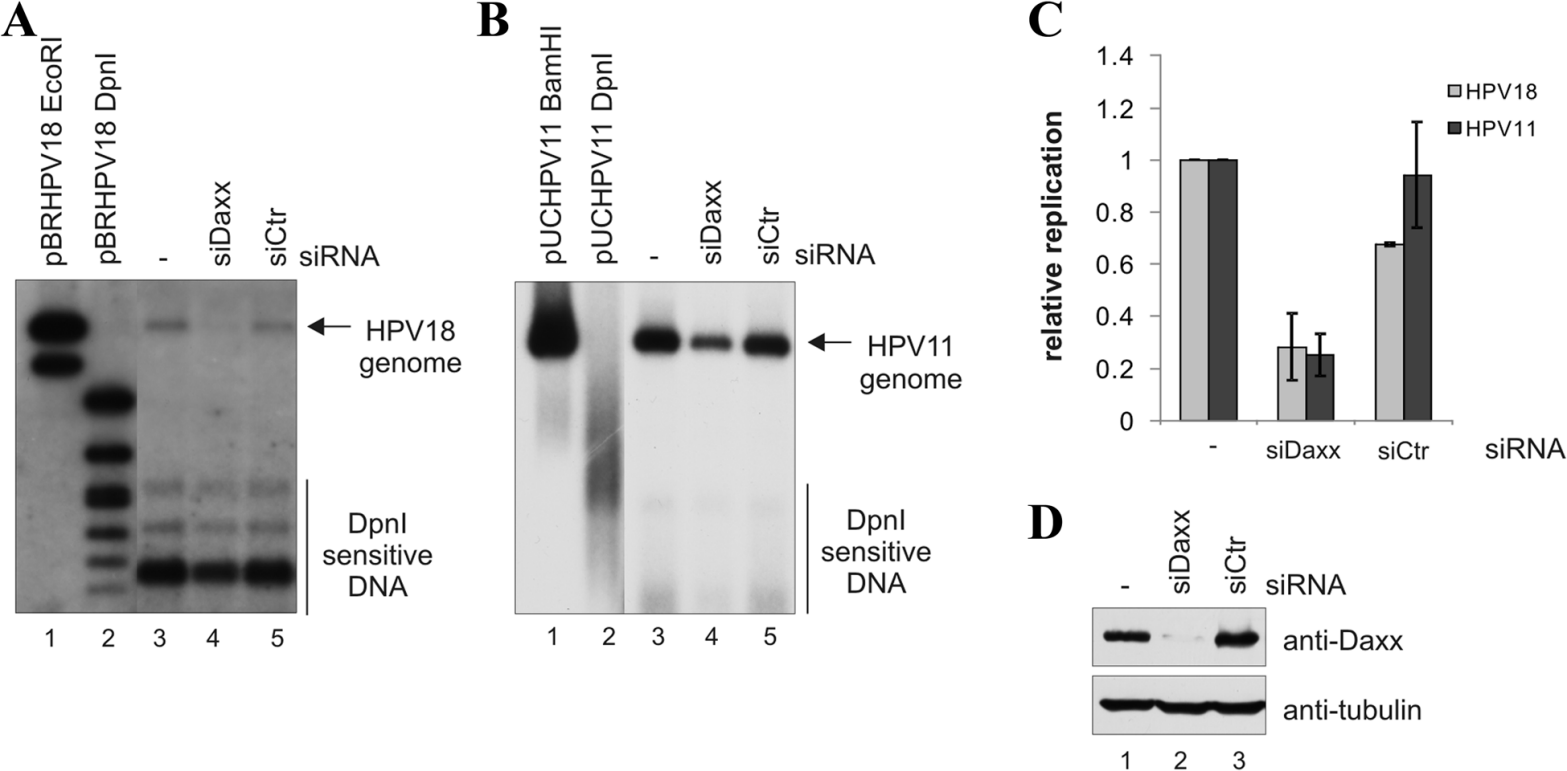
Down-regulation of DAXX represses HPV genome transient replication. U2OS cells were first transfected with Daxx siRNA or a control siRNA, and 24 h later with wt HPV18 (**a**) or wt HPV11 (**b**) genome. Cells were harvested 72 h after transfection, episomal DNA was isolated, digested with DpnI and linearizing enzyme, EcoRI or BamHI, and analyzed by Southern blotting using a radiolabeled probe specific for the viral genome. The linearizing enzyme (M1) and DpnI (M2) digested pBRHPV18 or pUCHPV11 plasmids as markers are shown on the right. **c** qPCR analysis of the episomal DNA isolated in replication assays. Episomal DNA was digested with DpnI and analysed by qPCR using HPV18 or HPV11 genome, and mitochondrial DNA specific primers. The data represent the average of three independent experiments and are presented graphically relative to the basal replication level of the HPV18 or HPV11 genome. **d** Western blot analysis of the DAXX protein levels in the cells used in replication assays. Cells (10^5^) were lysed, separated electrophoretically, and immunoblotted with anti-DAXX and anti-tubulin antibodies

In order to analyze the involvement of the DAXX protein in HPV genome replication in U2OS cells, we knocked down the expression of DAXX with siRNA and transfected the cells with wt HPV11 and wt HPV18 genomic DNA. Episomal DNA was isolated 72 h after transfection, and the newly replicated HPV11 and HPV18 viral DNA was analyzed by Southern blot (Fig 4a and b). Down-regulation of DAXX repressed the initial replication of both HPV11 and HPV18 genomes. We also analyzed the HPV DNA levels by quantitative real-time PCR, using mitochondrial DNA as an internal control. Consistent with Southern blot analysis, the quantitation of replication products by real-time PCR indicated that down-regulation of the DAXX protein reduced HPV DNA replication by 2-3-fold (Fig. 4c). The level of DAXX protein in U2OS cells treated with siDaxx or siCtr is shown in Fig. 4d.

### DAXX modulates HPV early gene expression in U2OS cells

**Fig. 5:**
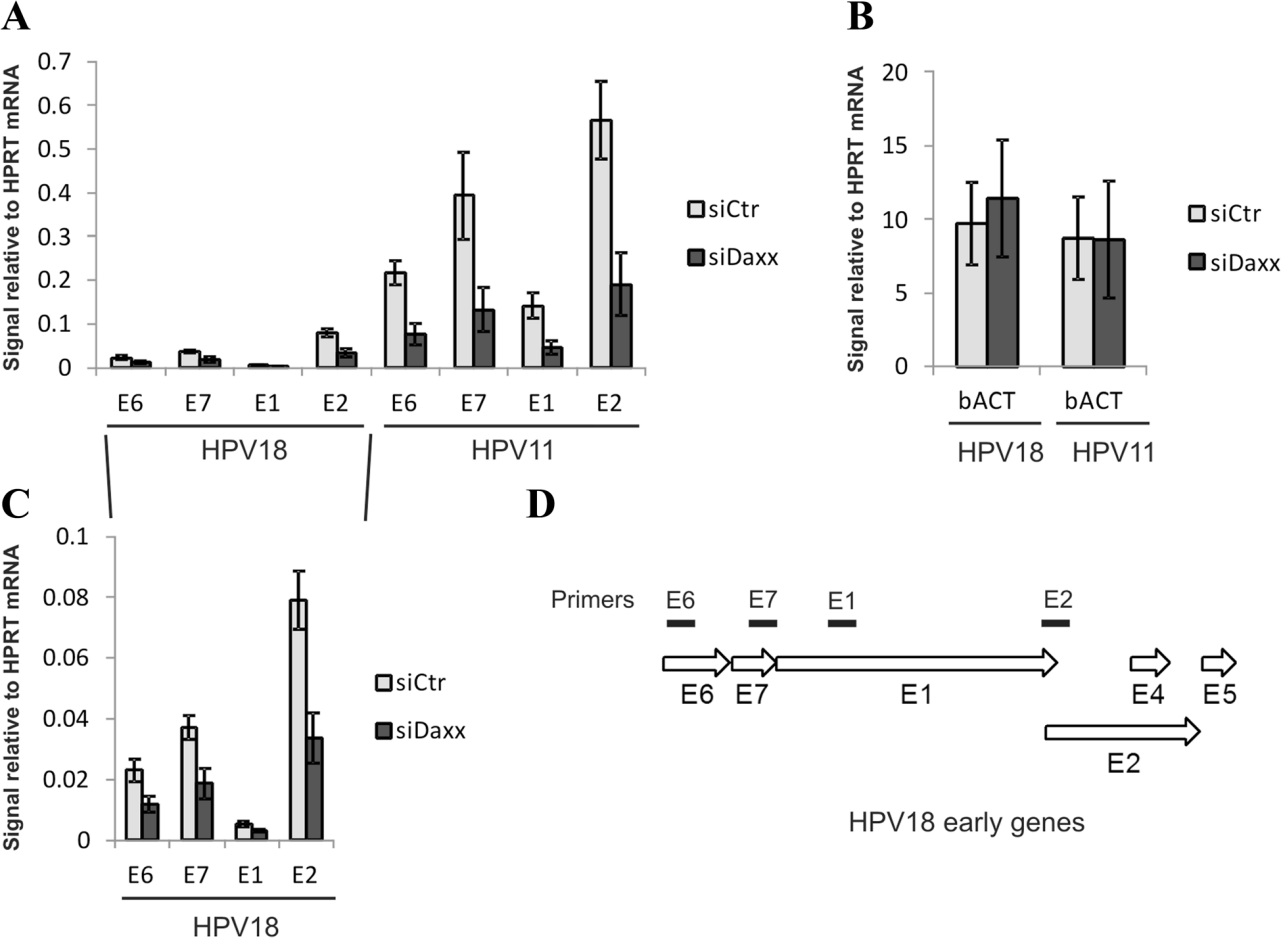
Down-regulation of DAXX represses HPV early gene expression. U2OS cells were first transfected with Daxx siRNA or a control siRNA, and 24 h later with wt HPV18 or wt HPV11 genome. 48 h after transfection total RNA was isolated, reverse transcribed and analyzed by qPCR. Each sample was analyzed in triplicate with E6, E7, E1, E2, beta-actin and HPRT1 specific primers. HPV11 and 18 (**a**) and bACT (**b**) mRNA levels were calculated relative to HPRT1 mRNA levels. The data represent the average of three independent experiments. Blow-out of HPV18 part from (**a**) is shown in (**c**). **d** The location of four different primer pairs targeted against HPV E6, E7, E1 and E2 coding sequences in HPV18 early region

The HPV genomes are transcriptionally active in U2OS cells and express all the viral early proteins required for initiation and establishment of HPV replication [19, 26]. In order to determine whether the DAXX protein is involved in papillomavirus early promoter regulation, we studied the expression of viral early genes that were transcribed from wt HPV18 and wt HPV11 episomes in U2OS cells. Four different primer pairs were used to measure the activity of HPV early promoters. E6, E7, E1 and E2 primers target the corresponding coding sequences in the viral genome (Fig. 5d). Most of these primers target multiple HPV transcripts in U2OS cells [26]. HPV18 E6 primers detect six differently spliced transcripts from early promoter P102; HPV18 E7 primers detect the same six transcripts, along with an additional three transcripts from promoter P520. HPV18 E1 primers detect a single E1 encoding transcript originating from promoter P102, and HPV18 E2 primers detect transcripts from three different promoters (P102, P811 and P1193). HPV11 primers were targeted to similar positions in the HPV11 genome.

U2OS cells were first transfected with siCtr or siDaxx, and twenty-four hours later, the cells were electroporated with 2 μg of either wt HPV11 or 18 DNA. After an additional 48 hours, RNA was extracted and the HPV transcript levels were analyzed. In order to compare the level of viral early promoters of HPV18 and HPV11 genomes, the viral transcript signals were expressed relative to cellular HPRT1 mRNA level. The HPV11 early promoters were more active when compared to HPV18 in U2OS cells, which is consistent with the replication efficiency of HPV 11 and 18 genomes in U2OS cells (Fig. 4). siDaxx treatment of U2OS cells prior to transfection with HPV18 DNA decreased the viral early gene expression by approximately 2-fold (Fig. 5a and c). Similar results were obtained with the HPV11 genome (Fig. 5a). The knock-down of DAXX did not influence the expression level of cellular housekeeping gene beta-actin (Fig. 5b). In summary, our data indicate that DAXX knock-down leads to a reduction in virus early gene expression.

## Discussion

In this study, we have examined the HPV replication foci in relation to cellular ND10 in U2OS cells. HPV replication foci, containing the viral genome, virus early proteins required for initiation of the replication, and cellular components ensuring the efficient propagation of the virus, are formed at specific sites in the nucleus. Our results indicate that a portion of HPV replication centers co-localize partially with components of the ND10, the DAXX and PML proteins. This is consistent with the work of Swindle et al. [4], who have previously shown that in C33A cells transfected with HPV11 viral ori sequence and E1 and E2 expression vectors, the viral proteins co-localize, at least partially, with PML in a portion of the cells. In addition, Rivera-Molina and others have demonstrated that HPV11 DNA/E2 protein complex recruits ND10 proteins, HPV DNA/protein complex co-localized with DAXX independently of PML, and with PML independently of the DAXX protein [27]. These data confirm that papillomavirus genomes replicate in close proximity to ND10.

Several other DNA viruses (herpes simplex virus type 1, simian virus 40, human adenovirus type 5) replicate and transcribe predominantly at close proximity to ND10 [28]. These similarities might originate from the requirement to control common cellular factors that participate in viral genome replication or transcription. Components of ND10 are interferon inducible transcriptional repressors, and are believed to form a nuclear defense mechanism against viruses. As a result, most DNA viruses have developed mechanisms to antagonize the restrictive function of ND10 [29]. In the case of papillomaviruses, however, the interaction with ND10 is controversial. The DAXX protein appears to have a positive role in HPV transcription and regulation. Our results show that knock-down of one component of ND10, the cellular DAXX protein, leads to reduced HPV11 and HPV18 early transcription and viral replication in U2OS cells. This result is consistent with a more detailed study by Stepp and others [7] of the roles of three components of ND10 in the early stages of HPV18 infection in primary human keratinocytes; PML, SP100 and DAXX. They revealed that PML and DAXX knock-down leads to a reduction in HPV18 transcription, while SP100 behaves as a repressor of viral infection. The presence of PML and intact ND10 is also associated with enhanced BPV1 early gene expression [5].

Recent work from different groups has demonstrated that HPV infection triggers the host cell DNA damage response (DDR) by activating the ATM-Chk2-dependent pathway [25, 30–32]. Activation of ATM is necessary for efficient, productive amplification of HPV [33]. Many of the DDR proteins that co-localize with HPV replication compartments also associate with PML and have the potential to reside in ND10 and, along with PML, relocate to sites of DNA damage (reviewed in [34]). The ND10 may therefore form a depot of cellular proteins that are involved in viral replication or transcription, and this could explain why the cellular environment around ND10 could be particularly advantageous for the papillomavirus during certain stages of the viral life cycle. The ND10 can act as DNA damage sensors that go through structural changes, increasing in their numbers in response to DNA damage [35]. Indeed, it has been shown that within the poorly differentiated basal and parabasal layers of the stratified epithelia, where the early stage of the viral life cycle takes place, the number of ND10 is increased in the presence of HPV genomes [6].

While the replication of HPV origin-containing plasmids can be reconstituted in many cell types by expressing viral replication proteins E1 and E2 from heterologous plasmids, most of the established human cell lines fail to support HPV genome replication [36]. Papillomaviruses have the capacity to establish long-term persistent infection in U2OS cells. This is achieved through the interplay between viral and cellular factors involved in virus gene expression and genome replication. We have shown that, similar to human keratinocytes, in U2OS cells the BRD4 protein is localized in HPV replication foci in the beginning of replication and is displaced when the foci increase in size. HPV replication centers in U2OS cells contain markers of homologous recombination similar to HPV31 foci in human keratinocytes [25, 32, 33]. All these data suggest that the HPV replication and cellular mechanism triggered by it, is similar both in human keratinocytes and in a model cell line U2OS.

## Conclusions

The analysis of HPV replication foci in U2OS cells indicated that HPV replication compartments overlap partially with the DAXX and PML proteins and the early replication is modulated by the DAXX protein. This further confirms that HPV replication has many common features in U2OS cells and primary human keratinocytes, suggesting that U2OS cell line is suitable for studying the basic mechanisms of papillomavirus replication.

## Materials and methods

### siRNAs

The siRNA target sequence for human Daxx (5′-GGAGUUGGAUCUCUCAGAA) has been published previously [37]. As a control siRNA (siCtr), non-specific siRNA (5’ CCCUGUCAGUAUUGAUAGAAA) was used. Both siRNAs were purchased from Sigma-Aldrich.

### Cell lines and transfections

U2OS cells obtained from the American Type Culture Collection (ATCC) (number HTB-96) were cultured in Iscove’s modified Dulbecco’s medium supplemented with 10 % of fetal calf serum. The transfections were carried out either by electroporation as described in [38], applying 180 V or by lipofection using Lipofectamine^TM^ RNAiMAX as a transfection reagent according to manufacturer’s protocol (Invitrogen).

### Replication assay

For transfection, the pBRHPV18 and pUCHPV11 plasmids [19, 30] were digested with EcoRI and BamHI to release the wt HPV18 and wt HPV11 genome, respectively; linear genomes were gel-purified, re-ligated and concentrated by ethanol precipitation [30]. 4 × 10^5^ of U2OS cells were seeded onto 60 mm dishes the day before transfection with 200 pmol of siDaxx and siCtr siRNA duplexes by Lipofectamine^TM^ RNAiMAX. Transfected U2OS cells were counted 24 h post-transfection and an even count of cells was transfected by electroporation with 2 μg of circularized HPV18 or HPV11 genome and incubated for another 72 h. The cells were collected, counted and low molecular weight DNA was isolated from an even amount of cells. 4/5^th^ of the recovered DNA was cut with DpnI, which digests only unreplicated DNA, and a linearizing enzyme (EcoRI or BamHI) and analyzed by Southern blot analysis as described in [20]. 1/65^th^ of the remaining DNA was subjected to qPCR analysis – DNA isolated from U2OS cells was cut with DpnI. The primers chosen for qPCR analysis contained a DpnI site in the amplified region so that only newly synthesized DNA was analyzed.

### mRNA analysis

2.5 × 10^5^ of U2OS cells were seeded onto 60 mm dishes the day before transfection with 200 pmol of siDaxx and siCtr siRNA duplexes by Lipofectamine^TM^ RNAiMAX. U2OS cells were counted 24 h post-transfection and an even count of cells was transfected by electroporation with 2 μg of circularized wt HPV18 or wt HPV11 genome and incubated for another 48 h. RNA was isolated using TRIZol® reagent (Invitrogen) according to the directions of the manufacturer. 5 μg of DNase I-treated total cellular RNA was used in reverse transcription reactions with oligo(dT)_18_ primers using First Strand cDNA Synthesis Kit (Fermentas). The reaction products were treated with RNase H (Fermentas). 1/40^th^ of the cDNA was used for analysis by qPCR.

### qPCR

Quantitative real-time PCR was performed using HOT FIREPol® EvaGreen® qPCR Mix (Solis BioDyne). All samples were analyzed in triplicate and the target sequences were amplified for 40 cycles using the following primer pairs:

- Replication assay
- HPV18 (ATAGGTTGGGCAGCACATAC and CAGTTCCGTGCACAGATCAG); HPV11 (TCCATTGCTGAACCTACTAC and CATTTTAAACAAGCGGAATCACCTTGCAG); mtDNA (CACTCCACGGAAGCAATATG and GTGTCGTGTAGTACGATGTC).
- mRNA analysis
- HPV18 E6 (TGCGGTGCCAGAAACCGTTG and GCTCGGTTGCAGCACGAATG), HPV18 E7 (GCCGAACCACAACGTCACAC and GCTGCTGGAATGCTCGAAGG), HPV18 E1 (AGCAGACAGCAACAGCAATG and CGCAGGAATTGCACTATTGG), HPV18 E2 (AGGAAGATGCAGACACCGAAG and GGCTGTCTATGTCTTTACTGTC), HPV11 E6 (AGCGTGTGCCTGTTGCTTAG and CGTGCCTTTCCCAATATGTG), HPV11 E7 (GGTGGACAAGGTGGACAAAC and ATGTCTCCGTCTGTGCACTC), HPV11 E1 (ACCGTGGCACGTACATTAGG and TAACGGTCTGGCGCGTTATC), HPV11 E2 (GAGCCACATCGGGTTACAAG and ACCGTTTGGGTGGTGTTAGC), HPRT1 (GACTTTGCTTTCCTTGGTCAGG and AGTCTGGCTTATATCCAACACTTCG), bACT (CTGGAACGGTGAAGGTGACA and CGGCCACATTGTGAACTTTG).

### Immunoblotting

For immunoblotting analysis, the cells were washed twice with PBS, detached with PBS-EDTA and lysed with SDS loading buffer. Material from equal number of cells was loaded on each lane, proteins were separated by SDS-polyacrylamide gel electrophoresis and transferred by a semi-dry blotting method to a polyvinylidene difluoride membrane (Millipore Corp). Membranes were incubated with rabbit anti-Daxx antibody (1:8,000; Sigma-Aldrich) and mouse monoclonal anti-tubulin antibody (1:20,000; Sigma-Aldrich); unconjugated primary antibodies were visualized with secondary anti-mouse and anti-rabbit IgG antibodies conjugated with horseradish peroxidase (1:10,000; Icosagen). Chemoluminescent signal was detected using ECL kit from Amersham Biosciences.

### Immunofluorescence analysis

HPV minicircle genomes were used in immunofluorescence analysis. The construction and production of minicircle HPV genomes has been described in [39]. U2OS cells were electroporated with 2 μg of wt HPV11 or HPV18E8- minicircle viral genomes and grown on microscope slides. 6 days after electroporation cells were fixed with 4 % paraformaldehyde for 10 min and IF analysis was performed as described in Reinson et al. [25]. IF was performed with the following primary antibodies using indicated dilutions: DAXX (D7810; Sigma-Aldrich) 1:100; PML (sc-966; Santa Cruz Biotechnology) 1:100; PML (sc-5621; Santa Cruz Biotechnology) 1:50; rabbit polyclonal antibodies BRD4B and BRD4C [40] at final concentration 10 ng/μl; HPV11 E2 (ab100968, Abcam) at final concentration 20 ng/μl; and rabbit polyclonal antibody HPV18 E1 [25] at final concentration 20 ng/μl. Secondary antibodies Alexa Fluor® 568 Goat Anti-Rabbit IgG (A-11011); Alexa Fluor® 568 Goat Anti-Mouse (A-11004); Alexa Fluor® 488 Goat Anti-Rabbit IgG (A-11008); and Alexa Fluor® 488 Goat Anti-Mouse (A-10680) were used at a dilution 1:1000 and were purchased from Invitrogen. Cells were placed under coverslips with SlowFade® Gold antifade reagent with DAPI (Invitrogen) and analysis was performed with *Zeiss* confocal microscope *LSM710* using 63x oil-immersion objective**.**

### IF-FISH analysis

U2OS cells were electroporated with 5 μg of wt HPV18 minicircle viral genomes [39] and grown on microscope slides. 6 days after electroporation cells were fixed and IF analysis followed by FISH was performed as described by Reinson et al. [25]. IF was performed with primary antibody anti-DAXX (D7810; Sigma-Aldrich) 1:100 and secondary antibody Alexa Fluor 568 Goat Anti-Rabbit IgG (A-11004; Invitrogen) 1:1000; HPV DNA was detected with HPV18 specific probe. Cells were placed under coverslips with SlowFade® Gold antifade reagent with DAPI (Invitrogen) and analysis was performed with *Zeiss* confocal microscope *LSM710* using 63x oil-immersion objective**.**
